# Regulation of GABA_A_ and Glutamate Receptor Expression, Synaptic Facilitation and Long-Term Potentiation in the Hippocampus of Prion Mutant Mice

**DOI:** 10.1371/journal.pone.0007592

**Published:** 2009-10-26

**Authors:** Alejandra Rangel, Noelia Madroñal, Agnès Gruart i. Massó, Rosalina Gavín, Franc Llorens, Lauro Sumoy, Juan María Torres, José María Delgado-García, José Antonio Del Río

**Affiliations:** 1 Molecular and Cellular Neurobiotechnology, Institute for Bioengineering of Catalonia, and Department of Cell Biology, University of Barcelona, Barcelona, Spain; 2 Centro de Investigación Biomédica en Red de Enfermedades Neurodegenerativas (CIBERNED), Madrid, Spain; 3 Division de Neurociencias. Universidad Pablo de Olavide, Sevilla, Spain; 4 Institute of Predictive and Personalized Medicine of Cancer, Badalona, Spain; 5 Centro de Investigación en Sanidad Animal (CISA), INIA, Valdeolmos, Madrid, Spain; The Research Center of Neurobiology - Neurophysiology of Marseille, France

## Abstract

**Background:**

Prionopathies are characterized by spongiform brain degeneration, myoclonia, dementia, and periodic electroencephalographic (EEG) disturbances. The hallmark of prioniopathies is the presence of an abnormal conformational isoform (PrP^sc^) of the natural cellular prion protein (PrP^c^) encoded by the *Prnp* gene. Although several roles have been attributed to PrP^c^, its putative functions in neuronal excitability are unknown. Although early studies of the behavior of *Prnp* knockout mice described minor changes, later studies report altered behavior. To date, most functional PrP^c^ studies on synaptic plasticity have been performed *in vitro*. To our knowledge, only one electrophysiological study has been performed *in vivo* in anesthetized mice, by Curtis and coworkers. They reported no significant differences in paired-pulse facilitation or LTP in the CA1 region after Schaffer collateral/commissural pathway stimulation.

**Methodology/Principal Findings:**

Here we explore the role of PrP^c^ expression in neurotransmission and neural excitability using wild-type, *Prnp* −/− and PrP^c^-overexpressing mice (Tg20 strain). By correlating histopathology with electrophysiology in living behaving mice, we demonstrate that both *Prnp* −/− mice but, more relevantly Tg20 mice show increased susceptibility to KA, leading to significant cell death in the hippocampus. This finding correlates with enhanced synaptic facilitation in paired-pulse experiments and hippocampal LTP in living behaving mutant mice. Gene expression profiling using Illumina™ microarrays and Ingenuity pathways analysis showed that 129 genes involved in canonical pathways such as Ubiquitination or Neurotransmission were co-regulated in *Prnp −/−* and Tg20 mice. Lastly, RT-qPCR of neurotransmission-related genes indicated that subunits of GABA_A_ and AMPA-kainate receptors are co-regulated in both *Prnp* −/− and Tg20 mice.

**Conclusions/Significance:**

Present results demonstrate that PrP^c^ is necessary for the proper homeostatic functioning of hippocampal circuits, because of its relationships with GABA_A_ and AMPA-Kainate neurotransmission. New PrP^c^ functions have recently been described, which point to PrP^c^ as a target for putative therapies in Alzheimer's disease. However, our results indicate that a “gain of function” strategy in Alzheimer's disease, or a “loss of function” in prionopathies, may impair PrP^c^ function, with devastating effects. In conclusion, we believe that present data should be taken into account in the development of future therapies.

## Introduction

The cause of spongiform encephalopathy in Creutzfeldt-Jacob disease (CJD), scrapie in sheep or bovine spongiform encephalopathy (BSE) is an abnormal conformational isoform (PrP^sc^) of the *Prnp* gene product PrP^c^ [1]–[4]. Although early studies of the behavior of *Prnp* knockout mice described only minor changes [5], later studies reported that these mice develop an age-dependent impairment in memory consolidation, altered behavior and neurotransmission (see [6], [7] for reviews). Several authors reported that excitatory glutamatergic synaptic transmission, GABA_A_ receptor–mediated fast inhibition and late afterhyperpolarization were reduced or absent in mice lacking PrP^c^ [8]–[11]. However, other authors reported differences in inhibitory and excitatory neurotransmission between *Prnp −/−* and wild-type mice [12]–[16]. More recently, the function of PrP^c^ in the regulation of olfactory behavior and dendrodendritic synaptic transmission in olfactory neurons has been described [17]. Moreover, *Prnp −/−* mice show synaptic dysfunctions such as altered circadian rhythms and sleep [18], impaired hippocampal dependent spatial learning [19] and age-dependent impairment of memory consolidation [20]. Some of these functions such as memory consolidation are mediated by its receptor [21] and the stress-inducible protein 1 [22]. Here we explore the role of PrP^c^ expression in neurotransmission and neural excitability using wild-type, *Prnp −/−* and PrP^c^-overexpressing mice (Tg20 strain). By correlating neurohistopathology with electrophysiology in living behaving mice, we found that *Prnp −/−* mice but, more relevantly, Tg20 mice show increased susceptibility to KA, leading to relevant cell death in the hippocampus. This finding correlates with enhanced synaptic facilitation and hippocampal LTP in both types of mutant mice. Lastly, our study using Illumina™ microarrays and further validation with RT-qPCR demonstrate that genes encoding AMPA-kainate and GABA_A_-mediated receptors are co-regulated in *Prnp −/−* and Tg20 mice.

## Results

### Different KA sensitivity and severity of KA-induced seizures in Prnp −/−, and Tg20 with respect to wild-type mice

Mice were treated with KA for 2 h (4 i.p. injections and analyzed for additional 2 h). The onset and intensity of seizures induced by identical KA injections differed greatly between mutant (*Prnp −/−* and Tg20) and wild-type mice (Table 1). Although non-statistically significant, Tg20 mice showed a later onset of seizures (95.8±10.1 min; mean ± SEM) with respect to *Prnp −/−* mice (84.6±15.19 min). Wild-type mice showed few behavioral changes and only one wild-type mouse showed signs of Grade III seizures after 147 min, which corresponded to the hyperactivity stage (Table 1, see Experimental procedures for details). None of the wild-type mice died during the experiments. In contrast, one *Prnp −/−* and two Tg20 mice had severe seizures and died. The mean number±SEM of seizures in treated mice was as follows: Tg20 = 7.3±1.8, *Prnp −/−* = 14.8±0.9, and wild-type = 0.3±0.3. Although the number of seizures was low, all Tg20 mice showed Grade VI seizures, while *Prnp −/−* mice showed Grade V-VI. In addition, Tg20 had significantly longer seizures than *Prnp −/−* (33 min for Tg20 and 16 min for *Prnp −/−*).

**Table 1:** Effects of KA-induced status epilepticus and death in Tg20, *Prnp −/−*, and wild-type mice.

Mice genotype	Onset (min)	n° of seizures	Prioritary behavior
Tg20	60	17	VI (death)
Tg20	113	5	VI
Tg20	101	8	VI
Tg20	66	4	VI (death)
Tg20	121	5	VI
Tg20	114	5	VI
Wild-type	-	0	III
Wild-type	147	2	III
Wild-type	-	0	III
Wild-type	-	0	III
Wild-type	-	0	III
Wild-type	-	0	III
Prnp −/−	40	14	V–VI
Prnp −/−	118	13	V–VI
Prnp −/−	103	19	V–VI
Prnp −/−	120	15	V–VI
Prnp −/−	100	13	V–VI
Prnp −/−	27	Continue	V–VI (death)

The onset, the number of seizures, the behavioral stages and the time in the prioritary stage is shown in each case.

### Correlation between histological and behavioral effects after KA treatment in experimental mice

**Figure 1:**
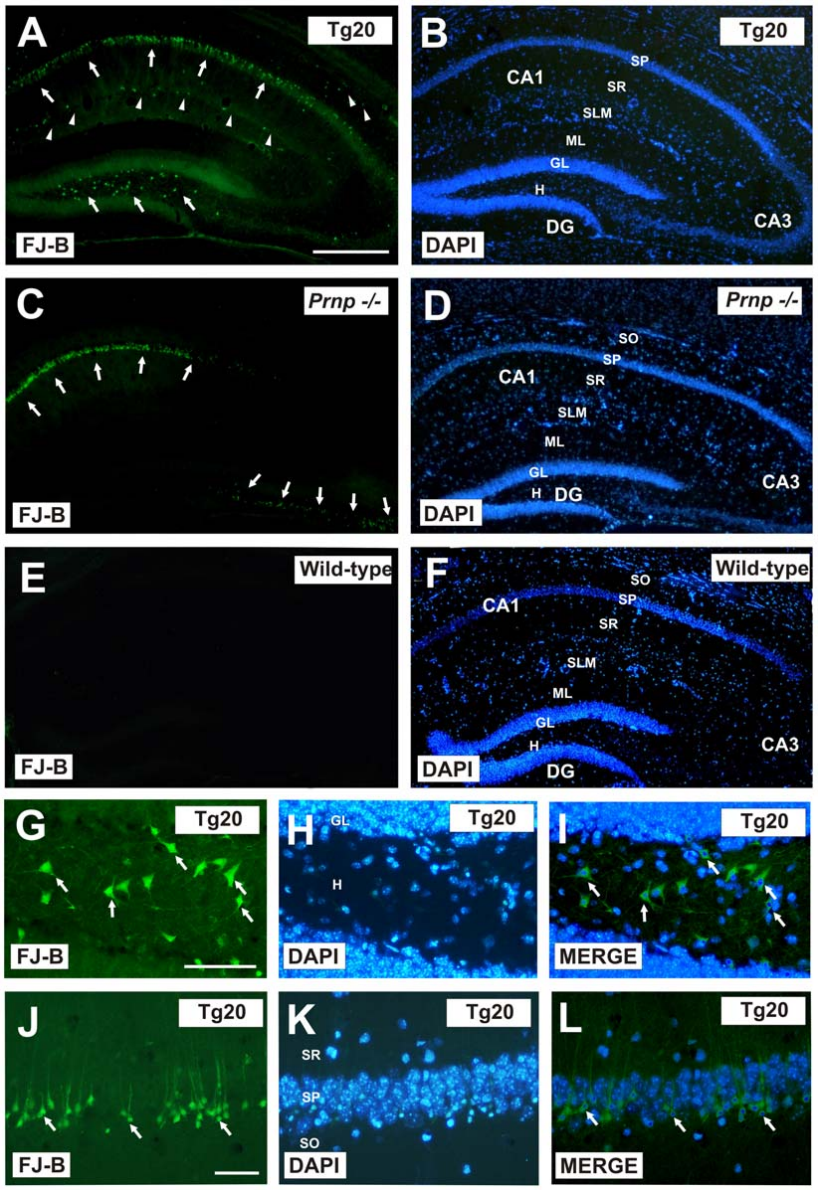
Increased seizure-related histopathology in *Prnp −/−,* Tg20 and wild-type mice. **A–F)** Low-power photomicrographs illustrating the pattern of Fluoro Jade-B (FJ-B) labeling (A,C,E) and DAPI (B,D,F) in the hippocampus of Tg20 (A–B), *Prnp* −/− (C–D) and wild-type (E–F) mice after KA injection. Dying pyramidal neurons (arrows in A and C) labeled with FJ-B were located in the CA1 and CA3 regions in Tg20 and *Prnp −/−* mice. However, a more widespread distribution of dying cells can be seen in Tg20 hippocampus (arrowheads in A) with hilar cells and subsets of interneurons in CA1-3 being intensely labeled. **G–L)** High-power photomicrographs illustrating the pattern of Fluoro Jade-B (FJ-B) labeling (G.J) and DAPI (H,K) in the hilus (G–I) and the CA1 (J–L) of Tg20 mice after KA injection. Numerous neurons labeled with Fluoro Jade-B (arrows in G,J and I,J) displayed condensed chromatin, as ascertained by DAPI staining (H–I, K–L) Abbreviations: CA1-3 hippocampal fields 1–3, DG, dentate gyrus; gl, granule cells; h, hilus; ml, molecular layer; sp; *stratum pyramidale*; sr, *stratum radiatum*; slm, *stratum lacunosum moleculare*; so, *stratum oriens*. Scale bars A = 250 µm pertains to B–F. G = 100 µm pertains to H–I; J = 150 mm pertains to I–L.

To determine whether the severity of changes in behavior observed in Tg20 and *Prnp −/−* after KA treatment correlated to neuron death in the hippocampus, we carried out Fluoro Jade-B (FJ-B) histochemical staining in coronal brain sections from KA-treated mice from all three experimental groups (Fig. 1). The pattern of cell death was different in each group. Tg20 mice showed more dying cells than *Prnp* −/− or wild-type mice. Although most dying cells were observed in the pyramidal layer of the CA1, with fewer in CA3 (Fig. 1), many FJ-B-positive cells were seen in the hilus of the dentate gyrus, the interphase *stratum lacunosum molecular*e/*stratum radiatum* (irl) and *stratum oriens* of the hippocampus proper of Tg20 (Fig. 1A, G). In contrast, dying cells were exclusively observed in the pyramidal layer of the CA1 and CA3 regions in *Prnp −/−* mice (Fig. 1C). Wild-type animals showed no cell death in the hippocampus after the KA-treatment (Fig. 1E). Neurodegeneration in Tg20 and *Prnp −/−* mice was accompanied by reactive astrogliosis in lesioned regions, determined by increased GFAP-immunostaining (Fig. S1) and ERK1-2 kinase activation in reactive glial cells (not shown).

### Recording of fEPSPs at the CA3-CA1 synapse in alert behaving PrP^c^ mice strains

**Figure 2:**
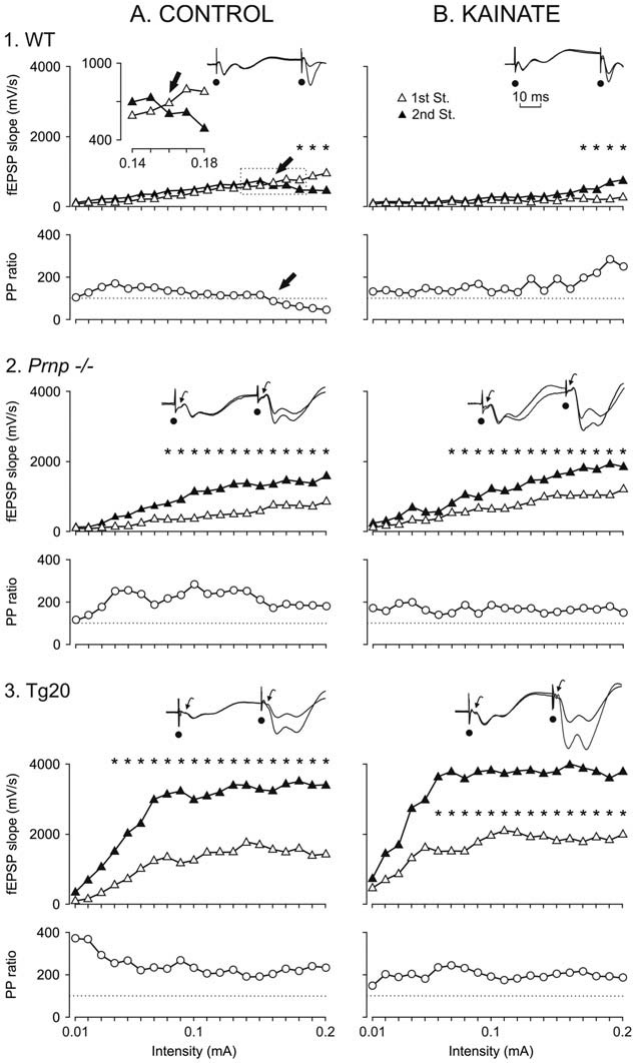
Input/output curves of the CA3-CA1 synapse using paired-pulse stimulation in wild-type (WT), *Prnp* −/−, and Tg20 mice before (*A*) and after (*B*) the administration of kainate (KA). **A)** Relationships between the intensity (from 0.01 to 0.2 mA) of pairs of stimuli (40 ms inter-stimulus interval) presented to Schaffer collaterals and the slope (in mV/s) of fEPSPs evoked at the CA1 layer, corresponding to the 1st (white triangles) and the 2nd (black triangles) pulses. Data were collected from WT (1.), *Prnp* −/− (2.) and Tg20 (3.) mice (n = 3 animals/group; n = 5 measurements/animal). The paired-pulse ratio was calculated as [(2nd/1st)×100] and it is illustrated for each group in the lower plot (white circles). Arrows in 1.WT indicate the intensity at which the paired-pulse evoked facilitation was reversed into depression . The inset in 1.WT is an enlargement of the dotted area, to illustrate the switch from paired-pulse facilitation to paired-pulse depression. Note that this reversal was not present in *Prnp* −/− or Tg20 groups, in which the second pulse was significantly larger than the first for most of the stimulation range. Representative averaged (n = 3) records of fEPSPs recorded in the CA1 area following paired (1st and 2nd St., at 40 ms inter-stimulus interval) stimulation of the ipsilateral Schaffer collaterals at two different (0.05 mA and 0.18 mA) intensities are illustrated for the three experimental groups. For comparative purposes, fEPSPs evoked by the 1st pulse were adjusted to the same amplitude. Dots indicate stimulus artifacts and bent arrows indicate the presence of afferent volleys. **B)** Same set of experiments as illustrated in A, but 30 min after a single injection of KA (8 mg/kg, i.p.). Note that KA did not significantly increase fEPSPs evoked by the 1st or the 2nd pulses in any of the 3 experimental groups. Asterisks indicate significant differences (*P*≤0.05) between fEPSPs evoked by the 1st versus the 2nd pulse.

Permanently implanted stimulating and recording electrodes in the hippocampus of behaving mice enabled us to follow the evolution of fEPSPs evoked at the CA3-CA1 synapse for several days in the same animal [23], [24]. During surgery, recording electrodes were oriented towards the apical dendrites of pyramidal CA1 cells (Fig. 2), as indicated by the negative fEPSPs recorded in most cases [24], [25]. The final location of recording and stimulating electrodes was checked histologically at the end of the experiments (Fig. S2).

### Input/output ratios were modified in Prnp −/−, and Tg20 with respect to wild-type mice

In a preliminary series of experiments, we measured the slope of fEPSPs evoked at the CA1 area by paired-pulse (40 ms inter-stimulus interval) stimulation of Schaffer collaterals at increasing intensities. In wild-type, (n = 3 animals; n = 5 measurements/stimulus intensity and animal), the slope of fEPSPs (in mV/s) evoked in the CA1 area by the first pulse increased steadily with current strength, to asymptotic values (at ≈950 mV/s) (Fig. 2). In contrast, fEPSPs evoked by the second pulse increased more or less in parallel with the fEPSPs evoked by the first pulse (but with ≈20% larger values) until a certain stimulus intensity (≈0.15 mA; see insert at Fig. 2A, 1.WT), after which the fEPSP slopes evoked by the second pulse were significantly smaller than those evoked by the first [F_(19,38)_ = 2.313; *P*<0.01, marked by asterisks in Fig. 2A, 1.WT]. Thus the paired-pulse facilitation evoked at low stimulus intensities in wild-type animals was reverted into a paired-pulse depression at high stimulus intensities. Indeed, as reported recently [26], the paired-pulse ratio [(2nd/1st)×100; see bottom graph at Fig. 2A, 1.WT] decreased steadily from facilitation to depression with an inflexion point at ≈0.15 mA (arrows in Fig. 2A, 1.WT). It has been suggested that the switch from facilitation to depression evoked by paired-pulse stimulation of increasing intensity is part of a protective and/or balancing homeostatic mechanism present in hippocampal synapses [27].

Input/output curves evoked at the CA3-CA1 synapse in *Prnp−/−* animals (n = 3 animals; n = 5 measurements/animal) were different in amplitude and profile from those evoked in wild-type mice. In the *Prnp −/−* group both the 1st and 2nd pulse evoked fEPSPs slopes greater than the corresponding values collected from wild-type (Fig. 2A, 2. *Prnp −/−*). Moreover, the slope of fEPSPs evoked by the 2nd pulse was significantly greater than those evoked by the 1st pulse over a wide range of stimulus intensities [0.08–02 mA; F_(19,38)_ = 4.123; *P*<0.001; Fig. 2A, 2. *Prnp −/−* upper graph]. More importantly, the paired-pulse ratio in *Prnp −/−* mice did not present a decreasing profile with the progressive increase in stimulus intensity (Fig. 2A, 2. *Prnp −/−* , bottom graph).

Finally, input/output relationships evoked at the CA3-CA1 synapse in the Tg20 group (n = 3 animals; n = 5 measurements/animal) were ≈3 times larger in fEPSP slopes than in the wild type group [F_(38,76)_ = 2.863; *P*<0.001; Fig. 2A, 1.WT vs. Fig. 2A, 3. Tg20 graphs]. Moreover, asymptotic values were reached at lower stimulus intensities (≈0.07 mA for the Tg20 group *vs*. ≈0.16 mA for the wild-type group). The slope of fEPSPs evoked by the 2nd pulse in the Tg20 group was significantly greater than those evoked by the 1st pulse over a wide range of stimulus intensities [0.05–0.2 mA; F_(19,38)_ = 4.517; *P*<0.001; Fig. 2A, 3. Tg20, upper graph]. Finally, the paired-pulse ratio in Tg20 mice showed a decreasing profile with the progressive increase in stimulus intensity, but always in the paired-pulse facilitation range (Fig. 2A, 3. Tg20, bottom graph). In summary, *Prnp −/−* and Tg20 mice presented significantly different input/output curves from wild-type, with a strong increase in paired-pulse facilitation and with the disappearance of the balancing homeostatic mechanism already described at CA3-CA1 in wild-type mice [26].

### Effects of a single KA injection on input/output relationships at the CA3-CA1 synapse in the three experimental groups

As reported above *Prnp −/−* and Tg20 mice showed increased sensitivity to KA-induced seizures. In order to discern the effects of a relative low dose of KA on input/output curves, we repeated the experiment illustrated in Fig. 2A, but 30 min after a single injection of KA (8 mg/kg, i.p.). KA injection produced slight changes in the profiles of input/output relationships evoked in wild-type, *Prnp −/−* and Tg20 animals (Fig. 2B). The paired-pulse depression evoked in the wild-type group disappeared at higher stimulus intensities (compare the upper graphs in Figs. 2A and 2B, for the wild-type group, 1.WT). Indeed, fEPSP slopes evoked by the 2nd pulse at high intensities in wild-type animals were significantly greater than the corresponding values for the 1st pulse [0.17–0.2 mA; F_(19,38)_ = 1.749; *P*≤0.05; Fig. 2A, B, 1.WT, upper graph]. No significant differences were observed between input/output curves evoked in *Prnp −/−* or Tg20 mice before or after KA injections. Thus, KA injection apparently disrupted the protective facilitation/depression mechanism evidenced by paired-pulse stimulation at increasing intensities [27], but did not affect fEPSPs evoked at the CA3-CA1 synapse in *Prnp* −/− or Tg20 mice, because this balancing mechanism is already disrupted and/or occluded in the latter animals.

### Prnp −/− and Tg20 mice presented more paired-pulse facilitation than wild-type mice

**Figure 3:**
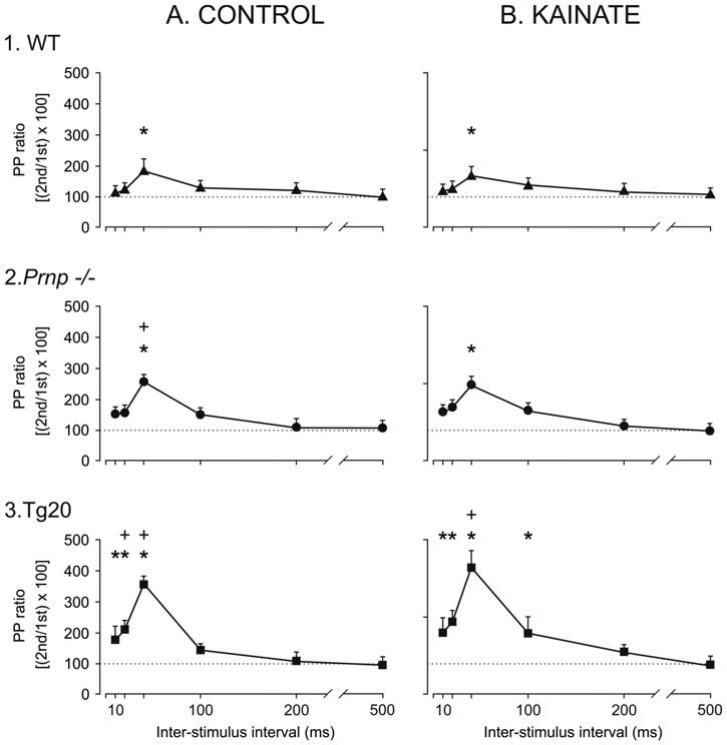
Effects of paired-pulse stimulation of the CA3-CA1 synapse at different inter-stimulus intervals for the three experimental groups before and after the administration of kainate (KA). **A)** Paired-pulse facilitation collected from wild-type (WT; 1, black triangles), *Prnp* −/− (2, black circles) and Tg20 (3, black squares) mice (n = 3 animals/group; n = 5 measurements/animal) in the control situation. Thus, each point indicates the mean value for 15 records ± SEM. **B)** Same set of experiments carried out 30 min after a single injection of KA (8 mg/kg, i.p.). Asterisks indicate significant differences (*P*≤0.01) between fEPSPs evoked by the 2nd pulse as compared with those evoked by the 1st, for the three experimental groups. The plus signs indicate significant differences (*P*≤0.001) between the amount of facilitation evoked in Tg20 and *Prnp* −/− groups as compared with the WT group.

The facilitation evoked by the presentation of a pair of pulses is a typical presynaptic short-term plastic property of some excitatory synapses, including the hippocampal CA3-CA1 synapses, and it has been correlated with neurotransmitter release [28]. As reported, recently, this contention can only be sustained at low stimulus intensities [26]. For this reason, we checked paired-pulse facilitation in the three groups of animals at different (10, 20, 50, 100, 200, and 500 ms) inter-stimulus intervals, but setting the stimulus intensity at 30–40% of the amount needed to reach asymptotic values [23], [29]. In this situation, wild-type animals were expected to present a larger fEPSP response to the 2nd (with respect to the 1st) stimulus at short intervals (<100 ms). Wild-type mice presented a significant [F_(5)_ = 54.810; *P*<0.01] increase in the response to the 2nd pulse at the 40-ms interval (Fig. 3A). *Prnp −/−* mice also presented significant paired-pulse facilitation at the 40-ms interval (*P*<0.01; Fig. 3A, 2. *Prnp −/−)*, whilst Tg20 mice presented significant paired-pulse facilitation at several (10, 20, 40) short inter-stimulus intervals (*P*<0.01; Fig. 3A, 3.Tg20). Paired-pulse facilitation at the 40 ms interval was significantly larger in *Prnp* −/− and Tg20 animals than in wild-type mice [F_(10,20)_ = 3.040; *P*≤0.01]. KA injection did not significantly modify paired-pulse facilitation in wild-type and *Prnp −/−* animals, but evoked a facilitation at medium (100 ms) inter-stimulus intervals in Tg20 mice [F_(10,20)_ = 4.507; *P*≤0.002]. In short, *Prnp −/−* and Tg20 presented more facilitation during the paired-pulse test than wild-type both in the absence and in the presence of a KA injection.

### LTP of the CA3-CA1 synapse is increased in Prnp −/− and Tg20 mice

**Figure 4:**
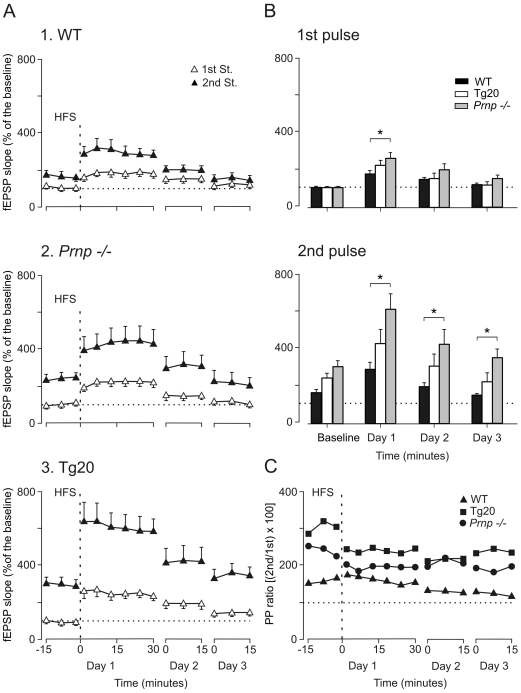
Evolution of fEPSPs evoked in the CA1 area by paired-pulse stimulation of Schaffer collaterals for the three experimental groups before and after a HFS session. **A)** Graphs illustrating the time course of changes in fEPSPs (mean ± SEM) following HFS stimulation of the Schaffer collaterals. The HFS train was presented after 15 min of baseline recordings, at the time marked by the dashed line and consisted of five 200 Hz, 100 ms trains of pulses at a rate of 1/s. This protocol was presented six times, at intervals of 1 min. fEPSPs are given as a percentage of the baseline (100%) slope. LTP evoked in WT (1.), *Prnp* −/− (2.), and Tg20 (3.) mice (n = 8 animals per group). The 100 µs, square, biphasic pulses used to evoke LTP were applied at the same intensity as that used for the single pulse presented following HFS presentation. The evolution of LTP was confirmed using a pair of pulses (1st, white triangles; 2nd, black triangles) with an inter-stimulus interval of 40 ms. Recordings were carried out for 30 min after the HFS session, and repeated for 15 min 24 h and 48 h later. **B)** Quantitative analysis of fEPSP evolution at the indicated times for the three experimental groups. Field EPSPs evoked by the 1st (top diagram) and the 2nd (bottom diagram) pulses are indicated separately. Asterisks indicate significant differences between groups (*P*≤0.01). **C)** Evolution of the paired-pulse ratio [(2nd/1st)×100] for WT (black triangles), *Prnp* −/− (black circles), and Tg20 (black squares) groups. Note that fEPSP slopes decreased across recording days for both 1st and 2nd pulses, but that their relationship [(2nd/1st)×100; white circles] increased steadily over time. Illustrated data correspond to mean ± SEM *, *P*<0.001, [F_(12,108)_ = 13.463] for differences between the 1st and 2nd pulses.

For the LTP study, animals were stimulated at Schaffer collaterals every 20 s for ≥15 min, until a stable fEPSP was recorded (baseline, Fig. 4A). The stimulus consisted of a double (100 µs, square, and biphasic) pulse presented at an interval of 40 ms [24]. Pulse intensity (30–110 µA) was set at 30–40% of the amount necessary to evoke a maximum fEPSP response [23]. For LTP induction, each animal was presented with a high-frequency stimulation (HFS) session consisting of five trains (200 Hz, 100 ms) of pulses at a rate of 1/s. This protocol was presented 6 times in total, at intervals of 1 min. In order to avoid evoking a population spike and unwanted EEG seizures, the stimulus intensity for HFS was set at the same amount used for generating the baseline record. After HFS, the same single stimulus used to generate baseline records was presented at the initial rate (3/min) for another 30 min. Field EPSPs were checked for a further 15 minutes, 24 h and 48 h after the HFS session (Fig. 4A). Since short-term potentiation seems to be dependent on the number of stimuli [30], paired-pulse evolution after HFS was monitored using a minimum number of paired stimuli per recording session (i.e., a maximum of 90 paired-pulses per recording day). Using this protocol, the three experimental groups presented a significant LTP lasting >24 h for both the 1st and the 2nd pulses [wild-type: F_(14)_ = 14.048, *P*<0.001. *Prnp −/−* : F_(14)_ = 0.982, *P*<0.001. Tg20: F_(14)_ = 12.509, *P*<0.001; Fig. 4A]. However, Tg20 presented significantly larger potentiation to the 1st and, mostly, to the 2nd pulse following the HFS session as compared with the wild-type [F_(6,42)_ = 1.388, *P*<0.01 for the 1st pulse and F_(6,42)_ = 1.423, *P*<0.01 for the 2nd pulse; Fig. 4B]. Values recorded in *Prnp −/−* mice were also higher (but not significantly) than wild-type.

It has been reported that HFS can modify the paired-pulse ratio, possibly as a result of competition with presynaptic release mechanisms [24], [30]–[32]. This suggestion was clearly confirmed here for HFS modified the paired-pulse ratio in Tg20 and *Prnp −/−* animals (Fig. 3C), but not in the wild-type group. A consequence of the latter result could be the rather small facilitation obtained in this group during baseline records. Moreover, HFS in *Prnp* −*/−* and mainly in Tg20 mice evoked a greater paired-pulse facilitation than in wild-type mice, even after LTP induction.

### Microarray analysis of gene expression and identification of canonical pathways by Ingenuity™ pathways analysis (IPA) in the three experimental groups

We used SAM software to identify genes that were differentially expressed in the untreated hippocampi of the three phenotypes. Thus, Tg20 mice had 336 genes that were de-regulated compared to wild-type mice. 207 of these were specific to Tg20 and the rest (129) were shared with *Prnp* −/− (Fig. S3). In addition, *Prnp* −/− showed 404 genes that were de-regulated in comparison with wild-type, of which 275 were specific to *Prnp* −/− and 129 were shared with Tg20 (Fig. S3). The 129 genes shared by *Prnp* −/− and Tg20 mice are co-regulated in the same way (up- or down-regulated) (Fig. S3). Next, we used the Web-based *on line* tool IPA to identify the set of biological pathways that were regulated in our experiment by integrating gene expression profiles using gene ontology (GO). As illustrated in Fig. S3, the list of significant canonical pathways containing co-regulated genes in Tg20 and *Prnp −/−* mice included: Protein Ubiquitination Pathways, Glycerolphospholipid Metabolism, Nitrogen Metabolism, GABA Receptor Signaling, D-Glutamine and D-Glutamate Metabolism, Glutamate Receptor Signaling, B-Cell Receptor Signaling, Hypoxia Signaling in the Cardiovascular System and Calcium Signaling. (Fig. S3).

### Differential expression of Glutamate and GABA_A_ receptors in prion mice strains

**Figure 5:**
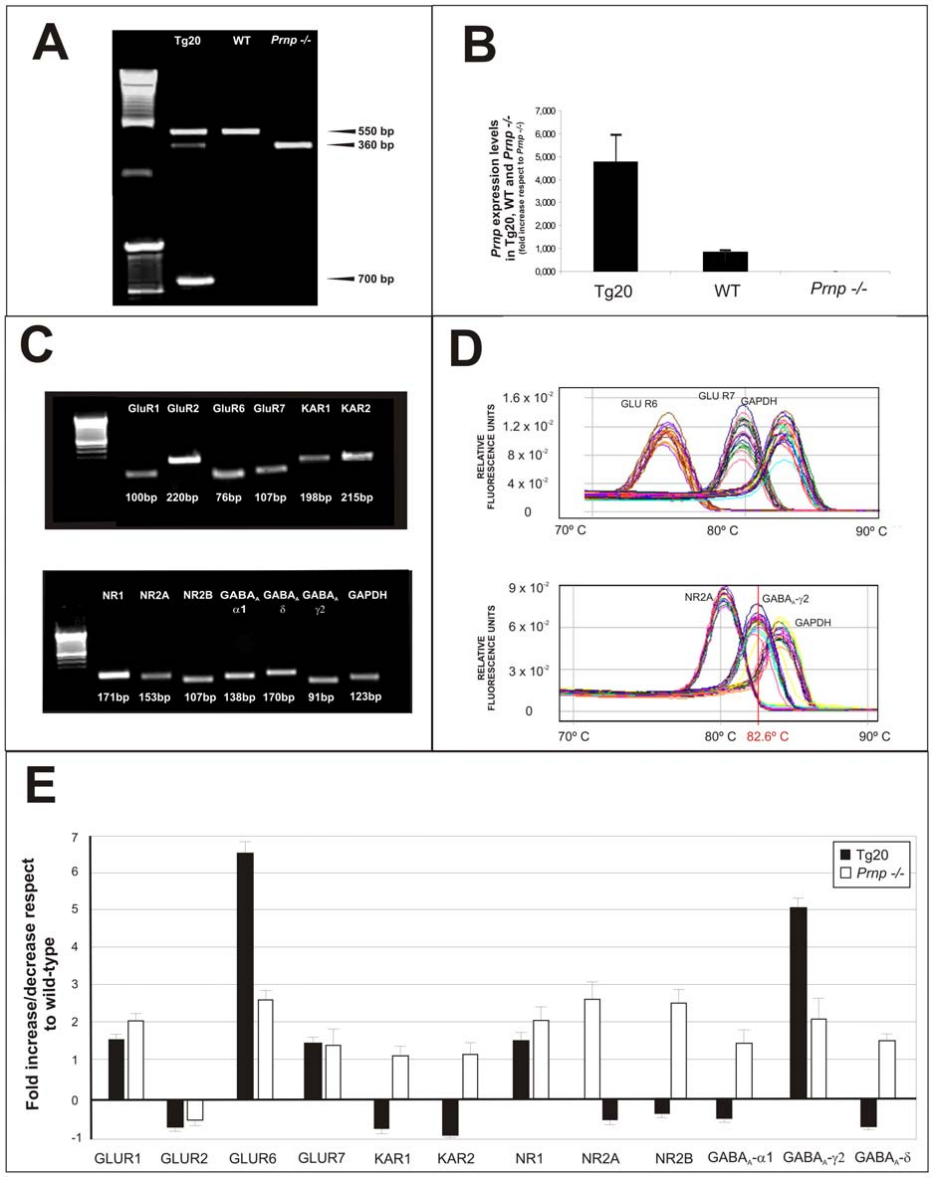
RNA expression of NMDA, AMPA, KA and GABA_A_ receptors in Tg20, *Prnp −/−* and wild-type mice. **A)** Agarose gel showing the amplified products of mice genotyping. **B)** Histograms showing the level of expression of *Prnp* mRNA obtained by RT-qPCR in each genotype (Tg20, *Prnp −/−* and wild-type). **C)** Agarose gel of the amplified products using the Sybr Green RT-qPCR primers for GluR1, GluR2, GluR6, GluR7, KAR1, KAR2, NR1, NR2A, NR2B, GABA_A_-α1, GABA_A_-δ, GABA_A_-γ2 and GAPDH. The size of the amplified bands is indicated in each lane in B. GADPH was used as internal control. **D)** Examples of the melting point analysis of the amplified products for GluR6, GluR7, NR2A, GABA_A_-γ2 and GADPH. Note the presence of different curves for each gene. The mean temperature for GABA_A_-γ2 is shown as an example. **E)** Histogram illustrating the quantitative results of the RT-qPCR experiment of target mRNA levels. Histograms represent the mean ± SEM of three independent experiments correlating receptor subunits/GADPH levels by using the 2^−ΔΔ*C*t^ method. Fold increases or decreases were calculated with respect to wild-type data.

Illumina Sentrix 6 mouse v2 beadarrays contain probes for some of AMPA-Kainate receptor subunits but, unfortunately not all subunits are represented (e.g., GluR6 and GluR7). In addition, any minor deregulation in specific genes cannot be determined since it may be masked in the hybridization procedure. However, GluR1 and GABA_A_-γ2 were among the 129 co-regulated genes. Thus we used RT-qPCR to extend the analysis to all glutamate and GABA_A_ receptors (Fig. 5). First, *Prnp* expression levels in the hippocampus were analyzed in wild-type and Tg20 samples and *Prnp −/−* mice by RT-pPCR (Fig. 5A–B). There was a 4.9 fold increase in *Prnp*-expression in Tg20 compared to wild-type whereas *Prnp* was not detected in *Prnp −/−* mice. Next, we analyzed the differential expression of AMPA-Kainate and NMDA receptor subunits (NR1, NR2A and NR2B) and GABA_A_ receptors subunits by RT-qPCR using specific primers (Table S1). GluR1 subunit was up-regulated 1.3 fold and 2 fold in Tg20 and *Prnp −/−* mice respectively compared to wild-type animals. In contrast, GluR2 was down-regulated in Tg20 (0.5 fold) and *Prnp −/−* (0.7 fold) compared to wild-type. GluR6 increased in Tg20 (6.5 fold) and in *Prnp* −/− (2.5 fold). In addition, GluR7 was also overexpressed in Tg20 and *Prnp −/−* (1.5 fold increase in both cases). Kainate receptor subunits 1 and 2 were up-regulated in *Prnp −/−* mice (1.2 fold increase (KAR1 and 2)) and down-regulated in Tg20 mice [0.62 (KAR1) and 0.3 (KAR2) fold respectively]. NR1 was up-regulated 2 fold in *Prnp −/−* and 1.5 fold in Tg20; NR2A and NR2B subunits were inversely regulated in Tg20 and *Prnp −/−* mice. NR2A was up-regulated 2.5 fold in Tg20 and down-regulated 0.7 fold in *Prnp −/−*. In contrast, NR2B was inversely regulated, with a 2.5 fold increase in *Prnp −/−* and a decrease (0.3 fold) in Tg20 an imals.

For GABA_A_ receptors, the receptor subunit α1 was overexpressed in *Prnp −/−* (1.4 fold increase) and down-regulated in Tg20 (0.7 fold) with respect to wild-type mice. The expression of GABA_A_-δ in mutant mice was similar to those seen in α1: down-regulated in Tg20 (0.62 fold) and over-expressed in *Prnp −/−* mice (1.4 fold). Finally, GABA_A_-γ2 was strongly up-regulated in Tg20 (4.5 fold increase) and increased in *Prnp* −/− (2 fold increase). We conclude that Tg20 and *Prnp −/−* animals overexpress GluR1, GluR6 and GluR7 and GABA_A_-γ2 subunits and down-regulate GluR2. In addition, GABA_A_-α1 and GABA_A_-δ as well as KAR1 and KAR2 are up-regulated in *Prnp −/−* but down-regulated in Tg20 compared to wild-type.

## Discussion

Most studies of the synaptic functions of PrP^c^ have compared *Prnp −/−* mice (Zürich 1 or Edinburgh strains) with wild-type animals (see Introduction). The results have highlighted the anti-oxidative function and neuroprotective roles of PrP^c^ (e.g., see [6], [7], [33], [34] for reviews). Previous studies have shown that *Prnp −/−* mice are more susceptible to KA injections than wild-type [35], [36]. Here we hypothesized that the overexpression of PrP^c^ would enhance cell survival by decreasing the susceptibility to KA observed in knockout mice.

In order to test this hypothesis, we compared Tg20, *Prnp* −/− and wild-type mice to examine the effect of *Prnp* dosage on KA-mediated neurotoxicity. Surprisingly, our results did not vindicate our hypothesis because increased *Prnp* dosage (4.9 fold increase) in Tg20 mice led to stronger reactions to KA-treatment (e.g., epileptic behavior and cell death) than those observed in *Prnp*−/− or wild-type mice. For this reason, we compared these new data with those obtained after electrophysiological recording at the hippocampal CA3-CA1 synapse of living behaving mice and with parallel RT-qPCR of glutamate and GABA_A_ receptors in the hippocampus of these mice after a microarray analysis. We found increased cell death in the hippocampus of both Tg20 and knockout mice after KA-treatment compared to wild-type mice. For this reason, in addition to the neuroprotective functions reported for PrP^c^ (e.g., [37], [38]), our results demonstrate that PrP^c^ levels play a crucial role in controlling neuronal homeostasis and cell survival. *Prnp* dosage should be maintained to a certain levels that, when modified, leads to dramatic neurological defects. In *Prnp −/−* mice the re-expression of a single allele of the *Prnp* gene rescues the KA-susceptible phenotype of *Prnp −/−* mice [35] and behavioral deficits [19]. For PrP^c^-deficient mice we can consider a “loss of function phenotype”. However, our results from Tg20 mice are more difficult to reconcile with current knowledge of PrP^c^ physiology. In addition, our data show that Tg20 mice are more susceptible to KA than *Prnp −/−*. Whether these unexpected results in Tg20 mice are attributable to a “loss of function,” or a “masking” effect of endogenous PrP^c^ roles by the increased *Prnp* over-expression, or a gain of “neurotoxic” properties by high PrP^c^ levels warrants further study.

Tg20 mice showed enhanced neurotoxicity in the hippocampal formation but especially in cell types that were not affected in KA-treated knockout mice, such as interneurons, or hilar cells. This indicates a gain of function process in terms of susceptibility rather than a loss of function phenomena, at least for certain hippocampal cells, since in KA-treated *Prnp −/−* hippocampus such cells are healthy. In this scenario, two results should be taken into account. First, transient over-expression of PrP^c^ in non-neuronal cell lines activates apoptotic stimuli and cell death [39]. Second, some mouse models overexpressing non-mutated forms of PrP^c^ [40]–[42] displayed neurodegenerative symptoms. Taken together, these results reinforce the notion that enhanced PrP^c^-overexpression may impair cell homeostasis *in vitro* and *in vivo*. Although we have not analyzed them in detail, these two results may be generated by different mechanisms. Overexpression of PrP^c^ may increase PrP^c^ levels at lipid rafts of the plasma membrane, which may alter intracellular signaling cascades or redox homeostasis, leading to caspase activation. Enhanced signaling by antibody-mediated PrP^c^-aggregation triggers cell death by increasing the generation of reactive oxygen species (ROS) [43], [44]. In contrast, in some PrP^c^-overexpressing mice, enhanced PrP^c^ presence may lead to abnormal PrP^c^ aggregation , which would contribute to the neural deficits reported [41].

To date, most functional PrP^c^ studies on synaptic plasticity have been performed *in vitr*o. To our knowledge, only one electrophysiological study has been performed *in vivo*—that by Curtis and coworkers. They reported no significant differences in paired-pulse facilitation or LTP in the CA1 region after Schaffer collateral/commissural pathway stimulation. However, they found reduced LTP in the CA1 of older *Prnp −/−* mice compared to wild-type [16]. Here, we report that young-adult Tg20, but not *Prnp −/−*, mice presented significantly larger potentiation to the 1st and, especially, to the 2nd pulse following HFS of the CA3-CA1 synapse, compared to wild-type. In contrast to the data reported by Curtis *et al.* (2003) in anesthetized animals, *Prnp −/−* mice presented more synaptic facilitation in the paired-pulse test than wild-type, a variation that could simply reflect the different functional (anesthetized vs. alert) states. On the other hand, a recent study by Powell *et al*., indicates that Ca^2+^ stores are altered in *Prnp −/−* mice, which may explain some of the phenotypes identified in *Prnp −/−* mice (i.e., weaker slow afterhyperpolarizations (AHP) in hippocampal neurons) [45]. This AHP defect is not caused by voltage-dependent calcium channels or calcium-activated potassium channels as previously suggested [9]. Since AHP is a natural mechanism that modulates successive action potential firing it is reasonable to expect higher susceptibility in *Prnp −/−* mice to epileptic treatments and synapse potentiation. Although an early study reported a role of PrP^c^ in the regulation of Ca^2+^ stores in synaptosomal fractions [46], and some pathological effects of aggregated synthetic prion peptides treatments have been associated with the endoplasmic reticulum [47], whether enhanced PrP^c^ expression alters intracellular Ca^2+^ stores in Tg20 mice is still unknown.

On the other hand, the enhanced excitability in Tg20 and *Prnp −/−* mice can be mediated by the differential expression of specific glutamate or GABA_A_ subunit receptors. Indeed GluR2 down-regulation observed in both mice is currently associated with high neuronal excitability (e.g., [48]). In addition, the up-regulation of GluR6 [49] and GABA_A_-γ2 [50] is also correlated with higher excitability. All these receptor subunits are co-regulated in Tg20 and *Prnp −/−* mice. However, the distribution of FJ-B-positive dying cells in the hippocampus is different in Tg20 and *Prnp −/−* mice, especially in CA3 and interneurons of CA1-3, and in hilar cells. These differences may reflect the particular distribution of the different glutamate receptors in the hippocampus and the specific participation of AMPA or kainate receptors as well as NMDA receptors in the KA-induced effects [51], [52]. For example, in addition to the co-regulation of GluR1-2 and Glur6-GluR7 subunits, *Prnp −/−* mice increased KAR1 and KAR2 receptor subunits compared to Tg20. In contrast, both mice displayed increased expression of NR1, but *Prnp −/−* displayed enhanced NR2B expression. The distribution of NR1 in the hippocampus closely follows the distribution of NR2A and NR2B [53], [54]. However, recent studies reported that NMDA receptors play special role in CA3 recurrent networks with relevant roles in seizure generation [55]. In addition, Gardoni and collaborators have recently reported that reduced NR2B expression impairs LTP in Schaffer collaterals without the participation of NR2A [56]. Thus, the specific overexpression of NR2B, together with KAR1 and KAR2 in *Prnp −/−* may participate in the enhanced seizures observed in the pyramidal layer of CA3 in *Prnp −/−* mice. However, the NMDA receptor subunit NR2D is only present in the *stratum oriens* of the CA1 and CA2, the *stratum lucidum* of CA3 and the inner third of the molecular layer of the dentate gyrus [53]. Although we did not explore NR2D levels in the present study, it has recently been described that PrP^c^ inhibits NR2D subunits [57], thus PrP^c^-deficient neurons showed enhanced and drastically prolonged NMDA-evoked currents [57]. This may also explain our results, since the participation of NMDA receptor in KA-induced cell death in hippocampus has also been demonstrated in *Prnp −/−* neurons [35].

Recent reports have shown that PrP^c^ decreases the extracellular levels of amyloid oligomers, which indicates that it might be a target for putative therapies in Alzheimer's disease [58]. However, our results show that a “gain of function” strategy to increase *Prnp* dosage in Alzheimer's disease or a “loss of function” in case of prionopathies to prevent PrP^c^ to PrP^sc^ conversion may impair natural function or PrP^c^ leading to devastating effects if natural expression levels are modified. In conclusion, we believe that present data should be taken into account in the development of future therapies.

## Materials and Methods

### Mouse strains

*Prnp −/−* mice bred from a C57BL6J/129, Ola/Sv background [5] were purchased from EMMA (Monterotondo, Italy) We backcrossed to C57BL/6J for at least 15 generations to obtain *Prnp* −/− and wild-type littermates. 22 *Prnp* −/− adult mice were used in the study. In addition, 19 C57BL6J littermates were also used. PrP^c^-overexpressing mice (Tg20 strain) were purchased from EMMA (Monterotondo). They were generated from *Prnp* −/− mice as described by Fisher *et al.* [59]. 28 Tg20 mice were used in this study. Experiments were carried out in accordance with the guidelines of the European Union (2003/65/CE) and current Spanish regulations (BOE 252/34367-91, 2005) for the use of laboratory animals in chronic studies. All experimental protocols were also approved by the respective local Ethical Committees.

### Mouse genotyping

Specific primers for *Prnp* genotyping were designed in our laboratory based on the original P10 and P3 primers described elsewhere [5]: P10-new: 5′-cataatcagtggaacaagccc-3′; P4-new: 5′-gctacaggtggataacccctc-3′; P3-new: 5′-gccttctatcgccttcttgac-3′. 40 cycling condition were: 45″ 95°C; 45″ 62°C; 1′ 72°C, followed by a final extension at 72°C for 5 min. For Tg20 mice, the transgene was detected by specific primers as indicated [60].

### Kainate injections and scoring of seizure severity

The C57BL6J strain is seizure resistant in comparison to other mouse genetic backgrounds [61], [62]. As indicated, *Prnp −/−* mice were generated on a C57BL6J/129, Ola/Sv background [5]. Non-lethal convulsive seizures were induced in wild-type mice by successive injections of kainate (KA) (Sigma, Saint Louis, Missouri, USA) dissolved in 0.1 M phosphate buffered saline (PBS) Ph = 7.4. Animals were weighed and injected i.p. with one pulse of KA (8 mg/kg b.w.) every 30 min for 2 h (making a total of four pulses). Injected animals displayed forelimb clonus, rearing and falling, or continuous tonic clonic seizures. They were observed for 4 hours after the first injection. Seizure intensity was evaluated as described elsewhere [63], [64]. After the first KA injections, affected animals became hypoactive and immobile (Grade I–II). After successive injections, hyperactivity (Grade III) and scratching (Grade IV) were often observed. Some animals (especially *Prnp −/−* and Tg20 mice) progressed to a loss of balance control (Grade V) and further chronic whole-body convulsions (Grade VI). The behavior known as pop-corn bouncing was included in Grade VI of the scale used in our study. Multiple doses of KA treatments were used to identify the threshold of KA-mediate seizures.

### Fluoro Jade-B staining of dying neurons in brain sections

Mice were perfused with phosphate buffered 4% paraformaldehyde, pH = 7.3, postfixed overnight in the same fixative, and cryoprotected in buffered 30% sucrose. Coronal sections (30-µm thick) were obtained in a freezing microtome and processed in parallel. Free-floating sections were rinsed for 2 hours in Tris 0.1 M, pH = 7.4, mounted and air-dried at room temperature overnight. Next day, sections were pretreated for 3 min in absolute alcohol, followed by 1 min in 70% ethanol and 1 min in distilled water. They were then oxidized in a solution of 0.06% KMnO_4_ for 15 min. Following three rinses of 1 min each in distilled water, the sections were incubated for 30 min in a solution of 0.001% Fluoro Jade-B (Chemicon, Temecula, USA) containing 0.01% of DAPI in 0.1% acetic acid. The slides were then rinsed in deionised water for 3 min each, dried overnight, rinsed in xylene and coverslipped with Eukitt™ (Merck, Germany). Sections were examined using an epifluorescent microscope with blue-violet excitation light set at 450 nm and 350 nm, respectively. Fluoro-Jade stained cells emit green light with an excitation peak at 480 nm and an emission peak around 525 nm.

### Electrophysiological experiments in behaving mice

A total of 24 (8 from each experimental group) male adults (3–5 months old; 25–35 g) were used in the electrophysiological study. Additional animals were used in a preliminary study of the stability of the recording and stimulating systems. Upon arrival in the laboratory, animals were housed in separate cages (n = 8 per cage), but they were switched to individual cages after surgery. Mice were kept on a 12 h light/dark cycle with constant ambient temperature (22±1°C) and humidity (55±8%). Food and water were available *ad libitum*.

### Surgery

Animals were anesthetized with 0.8–3% halothane (AstraZeneca, Madrid, Spain). The gas mixture was delivered through a small anesthesia mask (David Kopf Instruments, Tujunga, CA) connected to a calibrated Fluotec 5 (Fluotec-Ohmeda, Tewksbury, MA, USA) vaporizer at a flow rate of 1–4 L/min oxygen. Animals were implanted with bipolar stimulating electrodes in the right Schaffer collateral-commissural pathway of the dorsal hippocampus (2 mm lateral and 1.5 mm posterior to Bregma; depth from the brain surface, 1.0–1.5 mm [65]. A recording electrode was also implanted in the ipsilateral *stratum radiatum* underneath the CA1 area (1.2 mm lateral and 2.2 mm posterior to Bregma; depth from the brain surface, 1.0–1.5 mm). Electrodes were made of 50 µm, Teflon-coated tungsten wires (Advent Research Materials, Eynsham, UK), bared at their tips for ≈0.3 mm. The recording electrode was implanted in the CA1 area using as a guide the field potential depth profile evoked by paired (40 ms interval) pulses presented to the ipsilateral Schaffer collateral pathway. The recording electrode was fixed at the site where a reliable monosynaptic (≤4 ms of latency) CA3-CA1 fEPSP was recorded. In short, the electrical stimulation of Schaffer collaterals evoked an afferent volley into the CA1 area, usually appearing as a small triphasic (positive-negative-positive) potential, with a latency of ≈1.5–2 ms±0.5 ms (Figure 2). The afferent volley was followed, ≈2 ms later, by a large negative wave when recorded at the *stratum radiatum* (see ref. [23] for details). A 0.1 mm bare silver wire was affixed to the skull as a ground. All the wires were connected to a four-pin socket (RS-Amidata, Madrid, Spain). The socket was affixed to the skull by two small screws and dental cement. Further details are provided elsewhere [23], [24] Experimental sessions started one week after surgery.

### Electrophysiological recording procedures

Recording sessions were carried out with 3 animals at a time. Animals were placed in separate small (5×5×10 cm) plastic chambers located inside a larger Faraday box (30×30×20 cm). Extracellular recordings were made with a high impedance probe (2×10^12^ Ω, 10 pF) using differential amplifiers with a bandwidth of 0.1 Hz–10 kHz (P511, Grass-Telefactor, West Warwick, RI, USA). For input-output curves, the stimulus intensity of paired pulses presented to Schaffer collaterals was raised from 0.01 mA to 0.2 mA in steps of 10 µA (Fig. 2). The selected stimulus interval was 40 ms, because this interval presents maximum facilitation for the CA3-CA1 synapse [24]. In all cases, the pair of pulses of a given intensity was repeated 5 times with time intervals ≥30 s, to minimize interferences with slower short-term potentiation (augmentation) or depression processes [28]. To record paired-pulse facilitation at different (10, 20, 40, 100, 200, and 500 ms) inter-stimulus intervals (Fig. 3), pulse intensity (50–400 µA) was set at 30–40% of the amount necessary to evoke a maximum fEPSP response [23], [29]. To evoke long term potentation (LTP), we used an HFS session consisting of five 200 Hz, 100 ms trains of pulses at a rate of 1/s. This protocol was presented six times, at intervals of 1 min. Field EPSP baseline values were collected 15 min prior to LTP induction using paired (40 ms inter-stimulus interval) 100 µs, square, biphasic pulses, presented every 20 s. As indicated above, paired-pulse intensity for baseline recordings and after the HFS session was set at 30–40% of the amount necessary to evoke a maximum fEPSP response and at an inter-stimulus interval of 40 ms. In order to avoid evoking a population spike or unwanted EEG seizures, the stimulus intensity during the HFS train was that used to generate baseline records. After each HFS session, the same paired-pulse stimuli (40 ms inter-stimulus interval) were presented every 20 s for 30 min during the first LTP session and for 15 min on the following two days.

### Drug administration in electrophysiology procedures

KA was dissolved in 0.1 M phosphate buffered saline (PBS) at pH 7.4. KA was injected at a dose of 8 mg/kg, i.p., 30 min before input/output curves and the paired-pulse test.

### Electrode location in electrophysiological procedures

At the end of the experiments, mice were deeply re-anesthetized (sodium pentobarbital, 50 mg/kg), and perfused transcardially with saline and 4% phosphate-buffered paraformaldehyde (PFA). Selected brain sections (50 µm) including the dorsal hippocampus were obtained on a microtome (Leica, Wetzlar, Germany), mounted on gelatinized glass slides and Nissl stained with 0.1% Toluidine blue to determine the location of stimulating and recording electrodes.

### Analysis of the electrophysiological data

Field EPSPs and 1-volt rectangular pulses corresponding to stimulus presentations were stored digitally on a computer through an analog/digital converter (CED 1401 Plus, Cambridge, England), at a sampling frequency of 11–22 kHz and an amplitude resolution of 12 bits. Commercial computer programs (Spike 2 and SIGAVG from CED) were modified to represent EMG and fEPSP recordings. Data were analyzed off-line for quantification of CRs and fEPSP slope with the help of home-made representation programs [23]. Computed data were processed for statistical analysis using the SPSS for Windows package. Unless otherwise indicated, data are reported as the mean±SEM. Acquired data were analyzed using a two-way ANOVA test, with group, session, or time as repeated measure. Contrast analysis was added to further study significant differences.

### Expression profiling analysis of WT, Prnp −/− and Tg20 with Illumina™ bead arrays

To determine gene expression changes in the three mouse strains, we performed a global transcriptome analysis using Illumina Sentrix 6 mouse v2 bead arrays. Total RNA from 4 mice (biological replicate experiments of wild-type, *Prnp −/−* and Tg20) was extracted from hippocampi and genotype. RNA concentration was measured with a Nanodrop™, and RNA quality was assessed by Bioanalyzer™ with RIN (RNA integrity number) ranging between 8.5 and 10.0. For each sample, 200 ng of total RNA was reverse transcribed, amplified by *in vitro* transcription and labeled with biotin-UTP using the Illumina Total Prep RNA amplification kit (IL1791, Applied Biosystem/Ambion, Austin, TX, USA) following the manufacturer's instructions. Labeled sample quality was assessed by Nanodrop™ and Bioanalyzer™. After pre-heating to 65°C for 5 min 750 ng of biotinylated cRNA was hybridized in a BeadChip Hyb Chamber with rocking for 16 h at 58°C. On the following day, bead arrays were washed in Illumina™ washing solutions in a Hybex waterbath: first with static incubation for 10 min at 55°C in E1BC solution, followed by 10 rinses by dipping in the same solution and shaking 5 min at 90 r.p.m. in an orbital shaker; the next wash was by dipping 10 times in 100% ethanol and shaking 10 min at 110 r.p.m. in an orbital shaker; this was followed by another wash in E1BC solution, dipping ten times, followed by 2 min shaking at 90 r.p.m. Washed bead arrays were blocked in E1 buffer for 10 min in a rocking incubator and for a further 10 min with 2 ml of E1 buffer plus streptavidin-Cy3. The fluorescent reagent was removed with E1BC solution, dipping ten times, plus 5 min shaking at 140 r.p.m. Finally, bead arrays were dried by centrifugation for 4 min at 275 r.p.m., followed by scanning in the Illumina Beadstation. The Beadscan software generates *.tif images and extracts raw data as tabulated text files. The raw data were summarized per probe using BeadStudio software Gene Expression and the summary data file was processed using the PILLA Web interface tool (Lozano et al, unpublished) an implementation of the Lumi package [66] developed within the Bioconductor project in the R statistical programming environment [67]. Data were normalized using the RSN (robust spline normalization) method and the VST (variance-stabilizing transformation) method. The log2 intensities were media centered and log ratios were computed as differences in log2 intensities for each probe. The SAM (significance analysis of microarrays) two-class unpaired comparison test was applied with 100 permutations to detect significant differences in gene expression between treated and control conditions, initially setting the statistical significance at a false discovery rate of 5%, with an arbitrary absolute fold chance cutoff set at 1.2 [68]. Whole genome expressional data results were filtered, with criteria selection of 1.2 Fold Change and Q-value less than 5%.

### Microarray functional analysis

We used the Ingenuity Pathway Analysis (IPA) software. Annotation of expression data was performed with reference to a number of sources which include; NIH DAVID the Gene Ontology Consortium, Kyoto Encyclopedia of Genes and Genomes (KEGG) pathway [http://david.abcc.ncifcrf.gov, http://www.geneontology.org, http://www.genome.jp/kegg/pathway.html]. The IPA tool was used to identify the biological functions that were most significant to the data set. Canonical pathways were also identified from the IPA library; canonical pathways that were most significant to the data set were selected [69]. The significance of the association between the data set and the canonical pathway was measured in 2 ways: i) a ratio of the number of genes from the data set that map to the pathway divided by the total number of genes that map to the canonical pathway was calculated. ii) Fischer's exact test was used to calculate a *p*-value determining the probability that the association between dataset genes and the canonical pathway is significant.

### RT-qPCR

RT-qPCR was carried out using ABI PRISM 7700 sequence detection system equipment and power Sybr Green master mix (Applied Biosystems). Reaction volumes of 12.5 µl were used with 0.5 µM primers. Specific primers were taken from a database (http://pga.mgh.harvard.edu/primerbank/) based on the published sequences (see Table S1). Amplification conditions consisted of 2″ denaturation at 95°C; 15″ of annealing at 60°C; and 1 min elongation at 60°C for 40 cycles. The results were normalized by the expression levels of the housekeeping gene, *gapdh*, which was quantified simultaneously with the target gene. To this mixture, we added 1 µl of the serially diluted cDNA prepared from tissue. A melting point analysis was carried out to improve the sensitivity and specificity of amplification reactions detected with the Sybr Green I dye. Data were analyzed by SDS 1.9.1 Software (Applied Biosystems) following the 2^−ΔΔCT^ method [70]. The significance of differences was assessed using the Student's *t*-test and the Sigma Stat software.

## Supporting Information

Figure S1 Photomicrographs of the CA1 region showing the pattern of GFAP immunostaining in untreated Tg20 mice (A) and after KA treatment (B–C). GFAP immunoreactivity strongly increased in the CA1 of KA-treated Tg20 mice. In addition, GFAP-positive cells after KA-treatment displayed the classical morphology of reactive cells with tick cellular expansions (arrows in C). Abbreviations as in Fig. 1. Scale bars A = 100 um pertains to B. C = 50 um.
(5.21 MB TIF)

Figure S2 A diagram illustrating the experimental design of the electrophysiological experiments carried out in behaving mice. (A) Animals were implanted with bipolar stimulating electrodes (St.) oriented towards the right Schaffer collateral-commissural pathway of the dorsal hippocampus and with a recording (Rec.) electrode aimed at the ipsilateral stratum radiatum underneath the CA1 area. A bare silver wire was affixed to the skull as a ground. (B–C) Photomicrographs illustrating the location of stimulating (B) and recording (C) electrodes. Scale bars are 200 µm. Abbreviations: D, L, M, V, dorsal, lateral, medial, ventral; DG, dentate gyrus; Sub., subiculum.
(3.51 MB TIF)

Figure S3 Venn diagrams (A–C) and histogram (D) representing the functional microarray analysis between Tg20, Prnp −/− and wild-type mice using IPA software. In A–C the number of regulated (A); the down-regulated (B) and the up-regulated (C) genes as well as the genes shared between genotypes are shown. (D) Histogram illustrating the canonical pathways (X axis) including co-regulated genes with higher probability (indicated by the threshold (dashed line) of the −Log(p-value)). Squares and connecting lines between bars indicate the gene number tendency between functions.
(1.14 MB TIF)

Table S1 List of PCR primers used in the RT-qPCR validation.
(0.04 MB DOC)
