# Correction: Regulation of GABA_A_ and Glutamate Receptor Expression, Synaptic Facilitation and Long-Term Potentiation in the Hippocampus of Prion Mutant Mice

**DOI:** 10.1371/annotation/15a013f5-fe53-4003-a3fe-0c315d1a5e7c

**Published:** 2009-12-18

**Authors:** Alejandra Rangel, Noelia Madroñal, Agnès Gruart i. Massó, Rosalina Gavín, Franc Llorens, Lauro Sumoy, Juan María Torres, José María Delgado-García, José Antonio Del Río

The third author's name was spelled incorrectly. The correct name is: Agnès Gruart. The correct citation is: Rangel A, Madroñal N, Gruart A, Gavín R, Llorens F, et al. (2009) Regulation of GABA_A_and Glutamate Receptor Expression, Synaptic Facilitation and Long-Term Potentiation in the Hippocampus of Prion Mutant Mice. PLoS ONE 4(10): e7592. doi:10.1371/journal.pone.0007592

