# Supplementary material for: Regulation of GABAA and Glutamate Receptor Expression, Synaptic Facilitation and Long-Term Potentiation in the Hippocampus of Prion Mutant Mice
Source: PLoS One. 2009 Oct 26;4(10):e7592. doi: 10.1371/journal.pone.0007592 (PMC2763346; doi:10.1371/journal.pone.0007592)
Supplement: Table S1 — List of PCR primers used in the RT-qPCR validation. (0.04 MB DOC) [file pone.0007592.s004.doc]

| Gene | Forward Primer 5’-3’ | Reverse Primer 5’-3’ | Amplicon (bp) |
| --- | --- | --- | --- |
| GluR1 | CTCGCCCTTGTCGTACCAC | GTCCGCCCTGAGAAATCCAG | 100 |
| GluR2 | GTGTCGCCCATCGAAAGTG | AGTAGGCATACTTCCCTTTGGAT | 220 |
| GluR6 | ATCGGATATTCGCAAGGAACC | CCATAGGGCCAGATTCCACA | 76 |
| GluR7 | AGGTCCTAATGTCACTGACTCTC | GCCATAAAGGGTCCTATCAGAC | 107 |
| KAR1 | AAAGGCCAGAGGTCCAACTAT | CCCCTTCAGCATTAAGTATGGGT | 198 |
| KAR2 | ATAGTCGCCTTCGCCAATCC | GTGTCCGTGGTCTCGTACTG | 215 |
| NR1 | AGAGCCCGACCCTAAAAAGAA | CCCTCCTCCCTCTCAATAGC | 171 |
| NR2A | TGATGAACCGCACTGACCCTA | TGGGGATGAAAGTCTGTGAGG | 153 |
| NR2B | GCCATGAACGAGACTGACCC | GCTTCCTGGTCCGTGTCATC | 107 |
| GABA-_A_ α1 | AAAAGTCGGGGTCTCTCTGAC | CAGTCGGTCCAAAATTCTTGTGA | 138 |
| GABA-_A_ δ | ATCTGCCTGGTTCCATGATGT | AGCCATAGCTCTCTAGGTCCA | 170 |
| GABA-_A_ γ2 | AGAAAAACCCTCTTCTTCGGATG | GTGGCATTGTTCATTTGAATGGT | 91 |
| GAPDH | AGGTCGGTGTGAACGGATTTG | TGTAGACCATGTAGTTGAGGTCA | 123 |
